# A Practical Treatise on Diseases of Children

**Published:** 1874-03

**Authors:** 


### Books Reviewed.

*A Practical Treatise on the Diseases of Children.* By J. Forsyth Meigs, M. D., and William Pepper, M. D. Fifth Edition, Revised and Enlarged. Philadelphia: Lindsay & Blakiston, 1874.

The treatise of Meigs and Pepper on Diseases of Children has enjoyed a large share of the favor of the profession. The fourth edition met with a favorable reception at the hands of medical practitioners and became a book of constant reference and study. Although only four years have elapsed since its publication the call has been urgent for another edition. In the preparation of this volume the authors have taken pains to carefully revise the text of the formei edition so as to make it conform with the views of the present day.

Among the articles in which the greatest changes will be noticed are those on diseases of the heart, progressive muscular sclerosis, or pseudo-hypertrophic muscular paralysis, the treatment of scarlet fever and measles, variola and vaccine disease. Chapters have also been added in pulmonary emphysema, pneumo-thorax, retro-pharyngeal abscess, tonsillar affections, malarial fever and scrofula. The chapters of Nervous Disorders of Infancy and Childhood are well written and embrace the most important subjects under the head, we do not notice however any mention of the influence of a syphilitic diathesis, either inherited or acquired upon the nervous system.

The illustrative cases are printed in smaller type and the text condensed so that although a large amount of matter has been added, the present edition is increased only ninety pages in size over the former.

With the many additions, which have been made the treatise of Meigs and Pepper will rank as one of the most complete works upon the subject in the English language.

